# Cocaine Body Packing: A New Record

**DOI:** 10.7759/cureus.11728

**Published:** 2020-11-27

**Authors:** Ravina Tanna, Roxana Bostina, Geraint Lloyd, Nikhil M Patel, Johan Bastianpillai

**Affiliations:** 1 Surgery, East and North Hertfordshire NHS Trust, Stevenage, GBR; 2 General and Colorectal Surgery, Royal Berkshire Hospital, London, GBR; 3 Surgery, London North West University Healthcare NHS Trust, London, GBR

**Keywords:** body packing, cocaine toxicity, surgery, toxicology

## Abstract

We present a case of a 39-year-old man who was brought in by ambulance to the ED after ingesting 103 packets of cocaine prior to return to the United Kingdom (UK) from Holland. He presented with a persistent sinus tachycardia and mild abdominal pain but no evidence of peritonitis on examination. Contrast-enhanced CT showed widespread distribution of packets from the stomach to the sigmoid colon. He was taken to theater for emergency laparotomy and retrieval of the packets, which was done successfully without the need of any bowel resection. He was then discharged to police custody following a 10-day admission. This is the highest number of cocaine packets reported in the UK literature. This case report discusses the importance of a multidisciplinary approach in safely managing body packers who also present with signs of cocaine toxicity.

## Introduction

Cases of body packing of illicit substances for smuggling have been reported in the medical literature since 1971, and is a worldwide phenomenon [1]. Cocaine is the most frequently transported drug; the UK National Crime Agency reported seizing 122.9 tonnes of this Class A substance from 2017 to 2018 [2]. Body packing includes all methods of smuggling within the gastrointestinal tract and ‘packers’ usually swallow packets of illicit substances for transportation purposes. This differs to body pushing, whereby smugglers conceal the drugs within natural orifices such as the rectum, and body stuffing which involves rapid swallowing of the containers to avoid detection during a search by police or customs officers [3]. Cases of body packing have reportedly increased due to heightened border security after the events of 9/11 in New York. Evidence supporting various methods of management is largely retrospective and composed mostly of single-center experiences and individual case reports [4]. At present, there are no national guidelines on the emergency management of body packers in the UK. We present a case of the highest number of packets of cocaine ingested by a single patient who presented to a district general hospital in the UK, a total of 103, who also demonstrated signs of cocaine toxicity. This unique case report highlights the need for a multidisciplinary approach in managing patients that present with signs of cocaine toxicity after having ingested a large number of cocaine packets.

## Case presentation

A 39-year-old male was brought in by ambulance to the ED profoundly tachycardic. The patient had returned from Holland the previous day and admitted to ingesting 103 packets of cocaine wrapped in nylon for the intent of smuggling the packages. The ambulance crew reported that when they arrived at his house he was very anxious, agitated, and clammy. Interestingly, it was the patient’s own family who called the ambulance as they were concerned he was exhibiting unusual behavior. An electrocardiogram (ECG) was taken in the ambulance which showed isolated episodes of supraventricular tachycardia (SVT). He stated to the ambulance crew he had drunk half a can of beer that day and denied previous illicit drug use. Upon arrival, he was tachycardic at 170 bpm and had a raised respiratory rate of 28. A repeat ECG done on arrival in the ED showed a sinus tachycardia but with no signs of SVT as seen initially with the ambulance crew. On examination he was alert, had no focal neurology and his abdomen was mildly tender but soft. There was no evidence to suggest serious bowel pathology such as peritonism or perforation. He had vomited three times since returning to the UK but reported normal bowel movements. He denied any chest pain, palpitations, or seizures. In the way of past medical history, he suffered from hypertension but was on no regular medications.

Investigations

**Figure 1:**
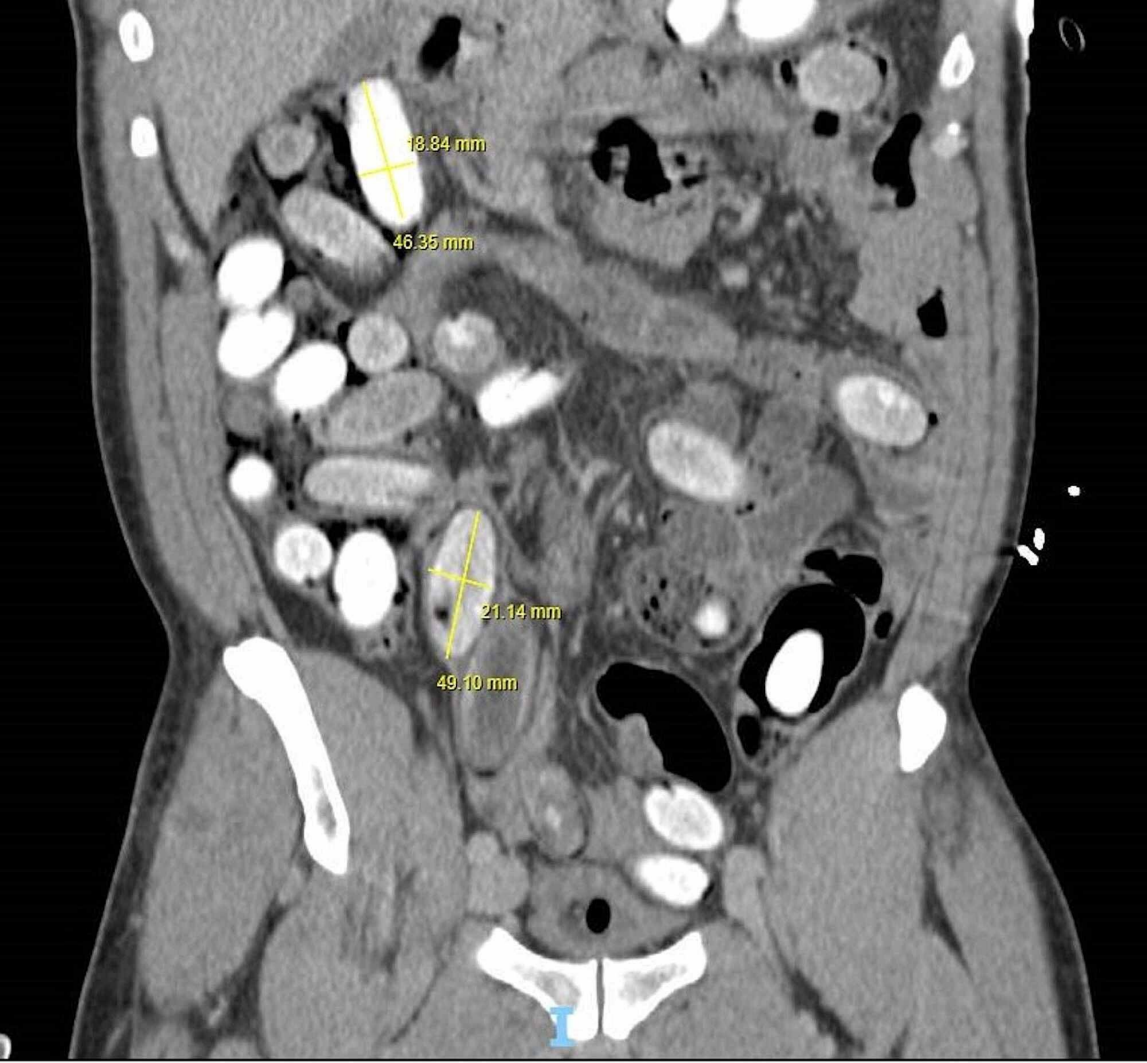
Coronal view of a CT scan showing the cocaine packets distributed in the abdomen.

**Figure 2:**
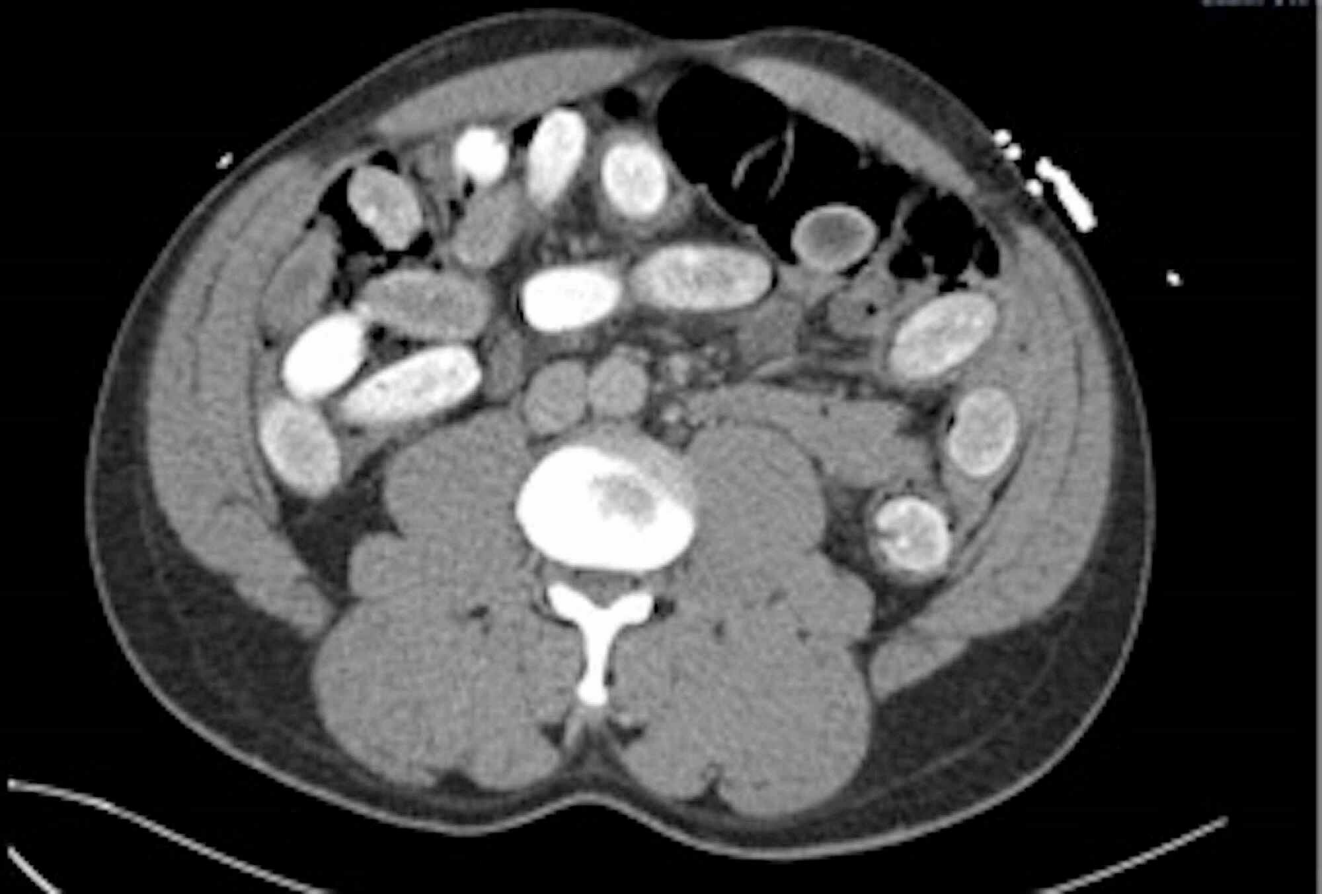
Axial view of a CT scan showing the cocaine packets distributed in the abdomen.

Initially, baseline blood tests were requested which were largely unremarkable apart from a mild acute kidney injury (AKI) with a creatinine of 158 (baseline 100) and a raised creatine kinase (CK) of 1061. He was sent for a CT abdomen and pelvis with contrast which revealed multiple ingested foreign bodies distributed throughout the stomach, duodenum, jejunum, and small bowel with further lesions present within the colon and rectum (Figures 1-2). 

 

There were no features of perforation, obstruction, collection, or free fluid in the abdomen. A venous blood gas (VBG) was taken and the initial VBG showed a pH of 7.30 and lactate of 7.6, which came down after IV fluids.

Treatment

When the patient first arrived and was being treated, the main concern was the persistent tachycardia for which 2 mg of lorazepam was administered intravenously. This was discussed with the cardiology team who advised to continue with lorazepam as there appeared to be no other abnormal ECG signs such as SVT. Multiple discussions were had regarding the best possible treatment plan for this patient. He was originally seen by the medical team and it was discussed whether an endoscopy would be appropriate. There was debate as to whether this was the safest treatment option and this was subsequently discussed with the Toxicology consultant on call who advised not to perform endoscopy due to risk of rupture of a cocaine packet. In this case, there was already suspicion of rupture due to the presenting clinical picture and the number of packets ingested. It was decided after surgical review to take the patient to the theater for an emergency laparotomy. His national emergency laparotomy audit (NELA) score revealed a mortality risk of 6.7% and morbidity of 74.5%. Intraoperatively, a midline incision was made and multiple packets were felt in the stomach, small bowel, and colon. It was necessary to make a gastrotomy, enterotomy in the terminal ileum and a colotomy in the cecum, transverse colon and sigmoid colon to remove all the packets. One packet was also found in the rectum. In total, 103 packets were removed with one seemingly partially ruptured. A size 20-Robinson drain was left in situ during closure. The total intraoperative time was roughly three hours. The patient was subsequently transferred to the ICU postoperatively.

Postoperative course

This patient was admitted to ICU postoperatively where he stayed for one night. His pain was controlled initially with a patient controlled analgesia (PCA) infusion pump and his urine output was satisfactory. He was extubated on day 1 postoperatively and stepped down to ward level care. He had a relatively slow postoperative recovery mainly due to pain and limited oral fluid intake. His C-reactive protein (CRP) began to rise on day 5 postoperatively and a CT abdomen and pelvis with contrast was requested which showed bibasal atelectasis but no collection or other abdominal pathology. By day 6 he had passed stool and was tolerating a soft diet. He was discharged on day 10 back to police custody. He returned once a week later for a dressing change where his wound was reviewed.

## Discussion

From our review of the current literature, whilst there are no national guidelines available, there are widely agreed principles with regard to the investigation and management of body packing cases. Some centers have created local guidelines where they have experienced a high throughput of cases [5]. Management is multimodal and ranges from simple laxatives and mechanical bowel preparation to a more invasive approach involving endoscopy and surgery. The factors that guide your approach are determined by the radiological evidence of the amount of packets, the location of these packets in the gastrointestinal tract, and the physiological state of the patient [1]. In most cases where a conservative approach has been used, the patient has shown no signs of toxicity and has ingested a relatively small number of packets compared to our case. Endoscopic retrieval tends to be reserved for cases where the packets have not passed the pylorus in a patient who is otherwise being managed conservatively [6]. Endoscopy as a sole means of managing these patients is only reported in those patients who have ingested a minimal number of packets due to the risk of rupture during the procedure, and is not widely reported in the literature. With regard to cocaine, body packers ingest on average 1 kg in quantity and the lethal dose of cocaine is between 1 and 3 g; therefore, rupture of even a single pellet can be fatal [7]. When the rupture of packet content occurs, intoxication can lead to cardiovascular instability and seizures. Other complications of body packing include mechanical obstruction of the alimentary canal or visceral perforation [8]. If this is suspected, an urgent surgical opinion is advised and it is likely a surgical approach would be deemed appropriate. Due to the associated mortality rate and risk of rupture of packets of illicit substances, all cases of body packing should be urgently investigated [9]. The literature predominantly describes plain abdominal radiograph (X-ray) and CT as the modalities of choice in the management of body packing; however, it has been reported that X-ray can yield significant diagnostic errors and ultimately leads to the need for a CT. Therefore, single abdominal low-dose CT has been deemed as the most useful diagnostic examination in body packing cases [10]. With the above in mind, in the case we present, the decision for an urgent laparotomy was made due to two main factors; the number of packets ingested and signs of cocaine toxicity. Endoscopic retrieval was too risky particularly as the packets were widely spread throughout the gastrointestinal tract and would not all be amenable to this method.

## Conclusions

This case report discusses the initial presentation and surgical management of a patient who presents with body packing. Due to the sheer amount of cocaine packets ingested and evidence of cocaine toxicity, the decision was made for overnight urgent surgical management due to the risk of cardiovascular instability predominantly. The need for multidisciplinary management of this patient was essential to ensure good patient outcomes.
